# Supplementary material for: The Influence of Cadmium on Fountain Grass Performance Correlates Closely with Metabolite Profiles
Source: Plants (Basel). 2023 Oct 29;12(21):3713. doi: 10.3390/plants12213713 (PMC10649124; doi:10.3390/plants12213713)
Supplement: Supplementary file 1 [file plants-12-03713-s001.zip › plants-2636814-supplementary.pdf]

Supplementary Material

The Influence of Cadmium on Fountain Grass Performance Correlates Closely with Metabolite Profiles

Zhaorong Mi[1,2], Pinlin Liu[1,2], Lin Du[1,2], Tao Han[1,2], Chao Wang[3], Xifeng Fan[3], Huichao Liu[1,2], Songlin He[1,2*] and Juying Wu[3,*]

[1] School of Horticulture and Landscape Architecture, Henan Institute of Science and Technology, Xinxiang 453003, China
[2] Henan Provincial Engineering Research Center of Horticultural Plant Resource Utilization and Germplasm Enhancement, Xinxiang 453003, China
[3] Institute of Grassland, Flowers and Ecology, Beijing Academy of Agriculture and Forestry Sciences, Beijing 100097, China
* Correspondence: hsl213@163.com (S.H.); wujuying@grass-env.com (J.W.); Tel.: +86-373-3040989 (S.H.); +86-10-51503285 (J.W.)

This file includes:

Figure S1: The morphological changes in leaf and root of fountain grass under Cd stress;

Table S1: The differential metabolites in roots of fountain grass and their relationships with leaf length, leaf fresh weight and leaf MDA content;

Table S2: The differential metabolites in roots of fountain grass and their relationships with root length, root fresh weight and root MDA content.

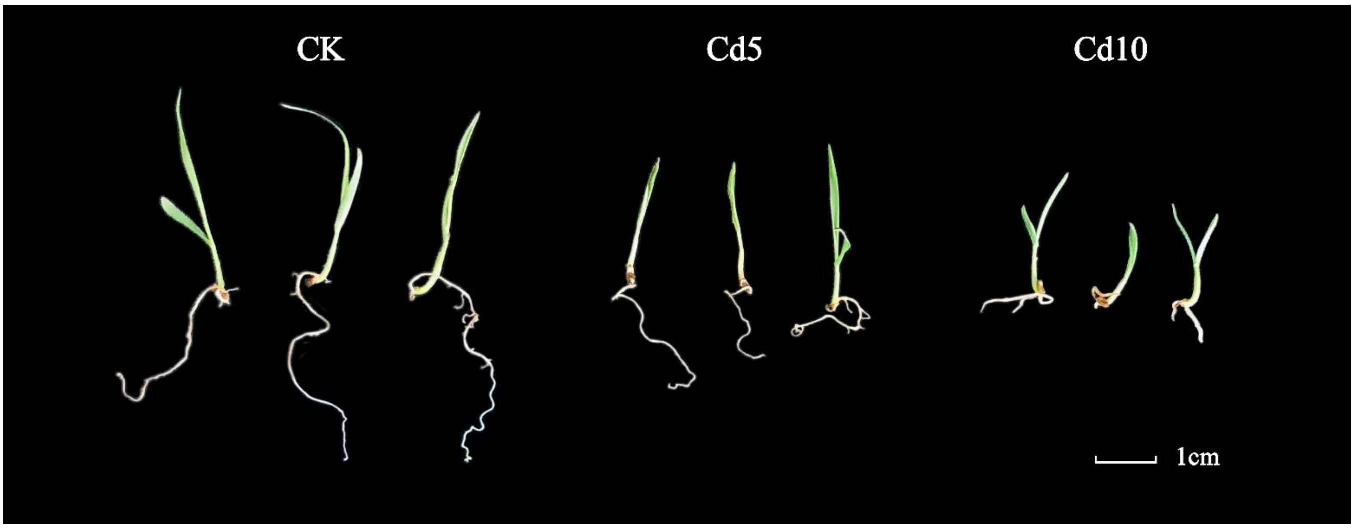

Figure S1. The morphological changes in leaf and root of fountain grass under Cd stress.

Table S1: The differential metabolites in leaves of fountain grass and their relationships with leaf length, leaf fresh weight and leaf MDA content.

Metabolite	Class	Fold Change	p	Leaf length		Leaf fresh weight		Leaf MDA	
				Coefficient	VIP	Coefficient	VIP	Coefficient	VIP
4-Guanidinobutyric acid	Amino acids	4.81	0.02	0.00	0.95	0.04	0.89	0.00	0.96
Gly-Leu	Amino acids	2.39	0.04	-0.01	0.94	-0.04	1.08	-0.01	0.90
D-ornithine	Amino acids	2.17	0.00	-0.03	1.09	-0.03	1.09	0.02	1.07
L-Asparagine	Amino acids	0.50	0.00	0.04	1.09	0.04	1.06	-0.02	1.06
Pantethine	Amino acids	0.49	0.02	0.05	0.96	0.01	0.80	-0.04	0.96
S-adenosyl-l-homocysteine	Amino acids	0.46	0.04	-0.02	0.90	0.03	0.97	-0.02	0.96
3-methyl-l-histidine	Amino acids	0.44	0.01	0.01	0.98	-0.01	0.83	-0.03	1.03
Cysteine-s-sulfate	Amino acids	0.40	0.00	0.02	1.07	0.02	1.09	-0.02	1.07
L-Histidine	Amino acids	0.38	0.01	0.01	1.03	0.05	1.15	-0.02	1.03
N-acetylhistidine	Amino acids	0.37	0.03	-0.02	0.91	-0.02	0.89	-0.02	0.97
Acetyl-dl-leucine	Amino acids	0.36	0.01	0.02	1.03	0.08	1.22	-0.02	1.01
L-Saccharopine	Amino acids	0.22	0.00	0.01	1.06	0.03	1.09	-0.03	1.08
Hydroquinone	Benzenoids	3.97	0.00	-0.04	1.08	-0.01	1.02	0.02	1.06
Pentamidine	Benzenoids	2.57	0.00	-0.04	1.10	-0.05	1.13	0.03	1.08
Aloin	Benzenoids	0.13	0.01	-0.01	0.98	-0.02	0.87	-0.03	1.05
D-Threitol	Carbohydrates	3.59	0.05	-0.02	0.89	0.06	0.76	-0.01	0.84
D-galacturonic acid	Carbohydrates	2.58	0.01	-0.04	0.98	0.06	0.73	0.04	0.99
Maltotriose	Carbohydrates	2.22	0.00	-0.03	1.08	0.01	0.99	0.02	1.07
Gentianose	Carbohydrates	0.45	0.03	0.03	0.96	0.11	1.08	-0.03	0.94
1-Deoxy-D-xylulose 5-phosphate	Carbohydrates	0.44	0.01	0.07	1.04	0.08	1.15	-0.03	0.96
Udp-d-glucose	Carbohydrates	0.43	0.02	0.01	0.96	-0.07	0.80	0.00	0.95
Perseitol	Carbohydrates	0.40	0.00	0.05	1.10	0.04	1.14	-0.02	1.06
N-acetyl-d-glucosamine 6-phosphate	Carbohydrates	0.27	0.00	0.00	1.02	0.01	0.99	-0.02	1.06
1-palmitoyl-sn-glycero-3-phosphocholine	Lipids	3.55	0.00	-0.05	1.06	0.00	0.97	0.03	1.04
Progesterone	Lipids	2.40	0.01	-0.05	1.01	-0.06	0.99	0.05	1.02
Cucurbitacin d	Lipids	2.23	0.02	0.01	0.93	0.04	0.85	0.02	0.98
11-beta-hydroxyandrosterone	Lipids	0.45	0.04	0.03	0.95	0.12	1.25	-0.02	0.90
Trans-traumatic acid	Lipids	0.43	0.03	0.03	0.97	0.07	1.16	-0.02	0.92
Oxandrolone	Lipids	0.43	0.05	0.04	0.95	0.12	1.26	-0.01	0.88
2-Isopropylmalic acid	Lipids	0.40	0.02	0.02	0.95	-0.03	0.89	-0.02	0.96
Endo-borneol	Lipids	0.31	0.04	-0.01	0.87	-0.08	0.59	-0.03	0.96
Mesaconic acid	Lipids	0.29	0.00	0.03	1.09	-0.01	1.00	-0.03	1.09
Guanosine 5'-monophosphate	Nucleosides	3.28	0.01	-0.04	1.02	-0.02	0.89	0.04	1.04
Adenosine monophosphate	Nucleosides	2.26	0.00	-0.02	1.05	-0.04	1.10	0.03	1.07
Guanosine diphosphate	Nucleosides	2.09	0.05	0.00	0.89	0.01	0.93	-0.01	0.86
Uridine 5'-monophosphate	Nucleosides	2.02	0.01	-0.05	1.08	-0.12	1.27	0.03	1.02
Guanosine	Nucleosides	0.36	0.03	-0.01	0.89	-0.07	0.64	-0.03	0.98
Oxalate	Organic acids	3.17	0.00	-0.05	1.04	0.00	0.88	0.04	1.03
Citrate	Organic acids	0.44	0.01	0.05	1.04	0.04	1.03	-0.01	0.98
L-Malic acid	Organic acids	0.43	0.00	0.03	1.07	0.07	1.18	-0.02	1.04
Glutaric acid	Organic acids	0.37	0.04	0.00	0.87	-0.08	0.57	-0.02	0.92
3-dehydroquinic acid	Others	4.19	0.00	-0.03	1.06	-0.06	1.14	0.02	1.04
Chlorambucil	Others	4.07	0.00	-0.03	1.09	-0.02	1.10	0.02	1.07
Astemizole	Others	2.08	0.03	-0.04	0.97	-0.15	1.29	0.03	0.94

Genistein	Phenylpropanoids and polyketides	4.83	0.02	-0.01	0.93	0.07	0.73	0.03	0.99
P-coumaric acid	Phenylpropanoids and polyketides	3.00	0.00	-0.04	1.08	0.00	0.96	0.03	1.08
3-hydroxy-4-methoxycinnamic acid	Phenylpropanoids and polyketides	2.01	0.02	0.00	0.97	-0.04	1.06	0.00	0.97
Indole-3-acetamide	Phytohormones	0.38	0.05	-0.03	0.85	-0.05	0.69	-0.02	0.95

Table S2: The differential metabolites in roots of fountain grass and their relationships with root length, root fresh weight and root MDA content.

Metabolite	Class	Fold Change	p	Root length		Root fresh weight		Root MDA	
				Coefficient	VIP	Coefficient	VIP	Coefficient	VIP
S-lactoylglutathione	Amino acids	16.06	0.04	0.00	0.84	0.00	0.88	0.00	0.86
4-Guanidinobutyric acid	Amino acids	7.61	0.00	-0.01	1.08	-0.01	1.09	0.01	1.07
Leu-Phe	Amino acids	5.45	0.00	-0.01	1.06	-0.01	1.05	0.01	1.06
Leucylleucine	Amino acids	4.56	0.01	-0.01	1.03	-0.01	1.03	0.01	1.03
Ala-Trp	Amino acids	4.49	0.00	-0.01	1.06	-0.01	1.05	0.01	1.07
Tryptophan	Amino acids	4.14	0.00	-0.01	1.04	-0.01	1.02	0.02	1.06
L-pipecolic acid	Amino acids	3.91	0.00	-0.01	1.01	-0.01	1.04	0.01	1.00
L-Glutamate	Amino acids	3.36	0.03	0.00	0.87	0.00	0.89	0.00	0.89
L-Arginine	Amino acids	3.28	0.01	-0.01	1.02	-0.01	0.99	0.02	1.04
L-Aspartate	Amino acids	3.23	0.00	-0.01	1.06	-0.01	1.06	0.01	1.04
O-acetyl-l-serine	Amino acids	2.78	0.03	-0.02	0.95	-0.01	0.95	0.01	0.90
N-acetyl-l-aspartic acid	Amino acids	2.78	0.05	0.00	0.80	-0.01	0.87	0.00	0.81
L-pyroglutamic acid	Amino acids	2.73	0.04	-0.01	0.93	0.00	0.89	0.02	0.96
3-Methylhistidine	Amino acids	2.58	0.01	-0.02	1.06	-0.02	1.04	0.01	1.04
D-ornithine	Amino acids	2.55	0.00	-0.01	1.06	-0.01	1.06	0.01	1.06
Alanine	Amino acids	2.55	0.01	-0.01	0.96	-0.01	0.99	0.00	0.94
Gamma-l-glutamyl-l-glutamic acid	Amino acids	2.51	0.00	-0.02	1.08	-0.01	1.06	0.01	1.06
gamma-aminobutyric acid	Amino acids	2.51	0.01	-0.01	0.97	-0.01	0.96	0.01	1.00
5-L-Glutamyl-L-alanine	Amino acids	2.43	0.02	-0.03	1.03	-0.02	0.97	0.02	1.00
DL-2-Aminoadipic acid	Amino acids	2.22	0.05	0.01	0.78	0.00	0.84	0.01	0.87
Gly-Leu	Amino acids	2.22	0.01	-0.01	1.00	0.00	0.99	0.01	1.04
Betaine	Amino acids	2.11	0.01	-0.01	0.99	-0.01	1.00	0.01	1.00
L-Valine	Amino acids	2.05	0.00	0.00	0.98	-0.01	1.02	0.01	0.99
Pantethine	Amino acids	0.36	0.00	0.01	1.04	0.00	1.04	-0.01	1.06
Allantoic acid	Amino acids	0.28	0.00	0.01	1.05	0.01	1.04	-0.01	1.07
Cysteine-s-sulfate	Amino acids	0.07	0.00	0.02	1.10	0.02	1.08	-0.01	1.07
Phenylethylamine	Benzenoids	4.77	0.03	0.00	0.86	0.00	0.89	0.00	0.89
Hydroquinone	Benzenoids	3.76	0.01	0.00	0.94	-0.01	0.98	0.00	0.93
Coniferyl alcohol	Benzenoids	3.30	0.00	-0.01	1.02	-0.01	1.03	0.01	1.04
Homovanillic acid	Benzenoids	0.49	0.00	0.02	1.09	0.01	1.06	-0.02	1.06
2-Hydroxyphenylacetic acid	Benzenoids	0.43	0.00	-0.01	0.95	0.01	1.00	-0.01	1.00
4-hydroxybenzyl alcohol	Benzenoids	0.39	0.00	0.01	1.08	0.01	1.07	-0.01	1.08
Aloeemodin	Benzenoids	0.38	0.01	0.01	1.03	0.02	1.04	-0.01	1.01

Benzamide	Benzenoids	0.29	0.00	0.02	1.10	0.01	1.08	-0.01	1.08
3-Hydroxybenzoate	Benzenoids	0.26	0.01	0.02	1.05	0.01	1.03	-0.01	1.01
Pyrocatechol	Benzenoids	0.22	0.04	0.03	1.02	0.03	0.97	-0.01	0.92
p-Hydroxyphenylacetic acid	Benzenoids	0.15	0.01	0.02	1.06	0.01	1.03	-0.01	1.01
Ribitol	Carbohydrates	2.50	0.01	0.00	0.96	-0.01	1.00	0.00	0.99
Galactinol	Carbohydrates	2.40	0.03	-0.01	0.93	-0.01	0.92	0.01	0.96
N-acetyl-d-glucosamine 6-phosphate	Carbohydrates	0.32	0.00	0.02	1.09	0.02	1.06	-0.01	1.05
2-deoxyribose 5-phosphate	Carbohydrates	0.31	0.00	0.02	1.08	0.01	1.06	-0.01	1.08
2'-Deoxy-D-ribose	Carbohydrates	0.29	0.01	0.00	0.94	0.00	0.96	-0.01	1.01
1-Deoxy-D-xylulose 5-phosphate	Carbohydrates	0.25	0.01	0.00	0.98	0.00	0.98	-0.02	1.02
Udp-d-glucose	Carbohydrates	0.22	0.05	0.04	1.01	0.02	0.95	-0.01	0.91
5(S)-HETE	Lipids	4.16	0.05	-0.01	0.93	-0.01	0.89	0.02	0.95
Stearidonic Acid	Lipids	3.68	0.00	-0.01	1.05	-0.01	1.04	0.01	1.07
4-oxoretinol	Lipids	3.10	0.01	-0.01	0.99	-0.01	0.98	0.01	0.99
Mesaconic acid	Lipids	2.62	0.01	-0.01	1.04	-0.01	1.01	0.02	1.05
Traumatic Acid	Lipids	2.25	0.01	-0.03	1.08	-0.02	1.03	0.01	1.02
3-hydroxy-3-methylglutaric acid	Lipids	2.17	0.01	-0.02	1.03	-0.01	1.01	0.01	1.01
Linoleic acid	Lipids	0.09	0.00	0.02	1.09	0.01	1.06	-0.01	1.06
Guanosine diphosphate mannose	Nucleosides	4.73	0.00	0.00	1.00	-0.01	1.03	0.01	1.00
S-adenosyl-l-homocysteine	Nucleosides	4.50	0.04	0.00	0.85	-0.01	0.88	0.00	0.88
Guanosine 5'-monophosphate	Nucleosides	3.25	0.01	-0.01	1.00	-0.01	0.98	0.01	1.03
Xanthine	Nucleosides	3.25	0.01	0.00	0.93	-0.01	0.97	0.00	0.92
Deoxyinosine	Nucleosides	3.03	0.04	0.00	0.87	-0.01	0.91	0.00	0.85
S-methyl-5'-thioadenosine	Nucleosides	2.46	0.01	0.00	0.97	-0.01	1.00	0.01	0.98
Uridine 5'-monophosphate	Nucleosides	2.11	0.00	0.00	0.98	-0.01	1.01	0.01	1.02
Thymidine 5'-diphosphate	Nucleosides	0.44	0.02	0.02	1.03	0.02	1.01	-0.01	0.99
Thymidine 5'-triphosphate	Nucleosides	0.41	0.01	0.02	1.03	0.01	1.02	-0.01	1.01
2'-deoxyguanosine 5'-monophosphate	Nucleosides	0.41	0.01	0.03	1.05	0.02	1.03	-0.01	1.00
Cytidine 5'-diphosphate	Nucleosides	0.40	0.01	0.02	1.04	0.02	1.03	-0.01	0.97
NADH	Nucleosides	0.38	0.03	0.02	0.97	0.02	0.95	-0.01	0.90
Flavine mononucleotide	Nucleosides	0.31	0.02	0.03	1.05	0.01	0.99	-0.02	0.98
2'-deoxyinosine	Nucleosides	0.27	0.00	0.00	0.99	0.00	1.02	-0.01	1.04
Adenosine	Nucleosides	0.24	0.00	0.02	1.10	0.02	1.08	-0.01	1.06
1-methyladenosine	Nucleosides	0.23	0.03	-0.02	0.82	0.00	0.88	-0.01	0.92
Deoxyadenosine	Nucleosides	0.22	0.01	-0.01	0.91	0.01	0.96	0.00	0.94
Adenosine 5'-monophosphate	Nucleosides	0.14	0.01	0.03	1.08	0.02	1.05	-0.01	1.02

Nicotinic acid adenine dinucleotide	Nucleosides	0.12	0.02	-0.01	0.88	0.01	0.92	-0.01	0.95
Glyoxylate	Organic acids	3.22	0.02	-0.02	0.98	-0.01	0.98	0.01	0.93
Glyceric acid	Organic acids	2.21	0.00	-0.01	1.04	-0.01	1.04	0.01	1.07
(R)-3-Hydroxybutyric acid	Organic acids	0.48	0.01	0.01	1.00	0.00	0.98	-0.01	1.00
Pyruvaldehyde	Organic acids	0.26	0.00	0.02	1.09	0.01	1.06	-0.01	1.05
Acetylcholine	Others	6.83	0.00	-0.01	1.05	-0.01	1.03	0.02	1.06
Betaine aldehyde	Others	6.45	0.00	-0.01	1.04	-0.01	1.02	0.01	1.05
3-dehydroquinic acid	Others	4.54	0.01	0.00	0.93	-0.01	0.98	0.00	0.93
Sphinganine	Others	3.66	0.02	0.00	0.90	0.00	0.92	0.00	0.92
Folinic acid	Others	3.21	0.00	-0.01	1.06	-0.01	1.04	0.02	1.06
Phytosphingosine	Others	2.69	0.03	0.00	0.86	-0.01	0.90	0.00	0.86
Nicotinate	Others	2.45	0.00	0.00	1.02	-0.01	1.05	0.01	1.03
Diacetyl	Others	2.16	0.02	0.01	0.85	0.00	0.92	0.00	0.92
Riboflavin	Others	0.44	0.03	0.01	0.95	0.02	0.97	0.00	0.91
Lusitanicoside	Others	0.38	0.00	0.01	1.03	0.01	1.06	-0.01	1.04
5-methoxyindoleacetate	Others	0.25	0.01	0.01	1.01	0.00	0.99	-0.02	1.02
Matairesinol	Others	0.25	0.00	0.01	1.02	0.01	1.02	-0.01	1.06
Bisdemethoxycurcumin	Phenylpropanoids and polyketides	23.63	0.00	-0.01	1.08	-0.01	1.08	0.01	1.08
3-hydroxy-4-methoxycinnamic acid	Phenylpropanoids and polyketides	9.31	0.01	-0.01	1.03	-0.01	1.00	0.02	1.05
P-coumaric acid	Phenylpropanoids and polyketides	8.76	0.00	-0.01	1.07	-0.01	1.08	0.01	1.08
Genistin	Phenylpropanoids and polyketides	6.12	0.00	-0.01	1.06	-0.01	1.04	0.02	1.07
Astilbin	Phenylpropanoids and polyketides	3.16	0.01	-0.01	0.97	-0.01	1.01	0.01	0.96
Neohesperidin	Phenylpropanoids and polyketides	2.95	0.03	-0.02	1.00	-0.02	0.95	0.02	0.97
Larixinic Acid	Phenylpropanoids and polyketides	2.44	0.04	-0.01	0.91	-0.01	0.89	0.01	0.95
7-methoxycoumarin	Phenylpropanoids and polyketides	2.37	0.00	-0.01	1.02	0.00	1.01	0.01	1.06
Naringin dihydrochalcone	Phenylpropanoids and polyketides	0.42	0.01	0.01	0.98	0.00	0.96	-0.02	1.01
Rutamarin	Phenylpropanoids and polyketides	0.33	0.00	0.00	0.99	0.00	1.02	-0.01	1.02
Diosmetin	Phenylpropanoids and polyketides	0.27	0.03	0.00	0.93	0.01	0.94	-0.01	0.96
3-(2-Hydroxyphenyl)propionic acid	Phenylpropanoids and polyketides	0.27	0.00	0.01	1.05	0.01	1.05	-0.01	1.07
Indole-3-butyric acid	Phytohormones	5.79	0.01	-0.01	0.98	-0.02	1.02	0.00	0.97

Phenylacetic acid	Phytohormones	5.08	0.00	-0.01	1.09	-0.01	1.08	0.01	1.08
Melatonin	Phytohormones	0.45	0.01	0.00	0.94	0.01	0.98	-0.01	0.98
Kinetin	Phytohormones	0.21	0.00	0.02	1.09	0.02	1.06	-0.01	1.06
